# Integrated WGCNA and PPI Network to Screen Hub Genes Signatures for Infantile Hemangioma

**DOI:** 10.3389/fgene.2020.614195

**Published:** 2021-01-15

**Authors:** Miao Xu, Tianxiang Ouyang, Kaiyang Lv, Xiaorong Ma

**Affiliations:** Department of Plastic and Reconstructive Surgery, Xinhua Hospital, Shanghai Jiao Tong University School of Medicine, Shanghai, China

**Keywords:** infantile hemangiomas, WGCNA, PPI network, AUC, hub genes

## Abstract

**Background:**

Infantile hemangioma (IH) is characterized by proliferation and regression.

**Methods:**

Based on the GSE127487 dataset, the differentially expressed genes (DEGs) between 6, 12, or 24 months and normal samples were screened, respectively. STEM software was used to screen the continued up-regulated or down-regulated in common genes. The modules were assessed by weighted gene co-expression network analysis (WGCNA). The enrichment analysis was performed to identified the biological function of important module genes. The area under curve (AUC) value and protein-protein interaction (PPI) network were used to identify hub genes. The differential expression of hub genes in IH and normal tissues was detected by qPCR.

**Results:**

There were 5,785, 4,712, and 2,149 DEGs between 6, 12, and 24 months and normal tissues. We found 1,218 DEGs were up-regulated or down-regulated expression simultaneously in common genes. They were identified as 10 co-expression modules. Module 3 and module 4 were positively or negatively correlated with the development of IH, respectively. These two module genes were significantly involved in immunity, cell cycle arrest and mTOR signaling pathway. The two module genes with AUC greater than 0.8 at different stages of IH were put into PPI network, and five genes with the highest degree were identified as hub genes. The differential expression of these genes was also verified by qRTPCR.

**Conclusion:**

Five hub genes may distinguish for proliferative and regressive IH lesions. The WGCNA and PPI network analyses may help to clarify the molecular mechanism of IH at different stages.

## Introduction

Infantile hemangioma (IH) is a benign tumor in children, and its rapid growth can lead to serious morbidity and even mortality (Zhu et al., 2018). It is estimated that 10% of infants have IH, and the frequency of hemangiomas in preterm infants with birth weight less than 1 kg increases to 22.9% (Blei, 2005).

The life cycle of hemangioma is divided into three stages. Samples from 6 months old infants were considered to be in the proliferative stage, while those from 24 months old infants were considered to be in the degenerative stage (Takahashi et al., 1994; Chang et al., 2008; Gomez-Acevedo et al., 2020). However, after tumor regression, 40–80% of IH will leave a permanent scar or a large amount of adipose tissue, especially in facial lesions, which can lead to deformity (Boscolo and Bischoff, 2009). Furthermore, although IHs can spontaneously regress, it is still difficult to predict the progression of some IHs. Therefore, some clinicians suggest that interventions for IHs should be performed at an early stage (Luo and Zhao, 2011).

Effective treatments for IHs include the use of corticosteroids and/or surgical removal of tumors (Chen et al., 2019). Although new advances have been made in treatment strategies for IHs, the main clinical problem remains the lack of reliable parameters to distinguish proliferative or regressive IH lesions.

At present, the etiology and pathogenesis of IH are not fully understood. In fact, there are differences in the expression of gene biomarkers in different stages of IH (Ritter et al., 2002; Greenberger et al., 2010a). P53 participated in the process of IH by regulating hypoxia-inducible factors and angiogenesis (Mabeta, 2018). In addition, immune cells are involved in the development of IHs through mesenchymal transformation of endothelium (Wu et al., 2017). Glucocorticoids affect IH growth by altering the “pro-angiogenic” environment of tumors, mainly by inhibiting high levels of vascular endothelial growth factor (VEGF)-A secreted by vascular endothelial cells (Greenberger et al., 2010b).

Although the history and progression of these lesions are clear, the etiology and exact mechanisms of occurrence and spontaneous degeneration remain unclear. A better understanding of the pathogenesis of hemangioma will provide innovative ideas for exploring more effective treatment strategies. Weighted gene co-expression network analysis (WGCNA) is a widely used method to build co-expression pairwise correlation matrices (Feltrin et al., 2019). Exclusively based on co-expression analysis will better represent genes with a small effect size acting together (Chaste et al., 2015). WGCNA provides a systems-level insight into the signaling networks that may be associated with a phenotype of interest (Liang et al., 2020). This study explored key genes and molecular mechanisms of tumor development by identifying differences of gene expression in IHs at different stages.

## Materials and Methods

### Data Collection

We collected IH related datasets from the gene expression omnibus (GEO) database. GSE127487 included gene expression profiling of skin tissue from six children of 6 months with hemangiomas, six children of 12 months with hemangiomas, six children of 24 months with hemangiomas and six healthy children. Matrix reports at the probe level were created through Average Normalization and background subtraction.

### Analysis of Differentially Expressed Genes

The difference of gene expression between IH and healthy children were analyzed by limma R software package (Ritchie et al., 2015). The differentially expressed genes (DEGs) were obtained using the criteria of *P* < 0.05 (Gu et al., 2020).

### Weighted Gene Co-expression Network Analysis (WGCNA)

The WGCNA of DEGs was performed using WGCNA R software package (Botia et al., 2017) to construct co-expression network. The soft-thresholding power was used as the correlation coefficient threshold. Then, module eigengene (MEs) with > 23 genes were selected using the dynamic tree cut method. In addition, the correlations between modules and clinical trait of patients were investigated using the Pearson correlation.

### Enrichment Analysis

To explore the potential biological roles of module genes, Gene ontology (GO) and Kyoto Encyclopedia of Genes and Genomes (KEGG) pathway enrichment were analyzed using clusterProfile R software package (Shi et al., 2018). Gene set enrichment analysis (GSEA) for module genes was performed with GSEA software (Reimand et al., 2019). *P* < 0.05 was chosen as the cutoff value.

### Protein–Protein Interaction Network

The selected module genes were analyzed by inputting them into the Search Tool for the Retrieval of Interacting Genes (STRING) database^1^. A combined score of ≥ 0.5 was considered as significant to construct a protein-protein interaction (PPI) network. The PPI network was displayed by Cytoscape software (Shi et al., 2018). The hub genes were chosen based on a higher number of associations with other genes.

### Quantitative Real-Time Polymerase Chain Reaction

A total of nine hemangioma skin tissue samples (three samples for 6 months, three samples for 12 months and three samples for 24 months) and three normal skin tissue samples were collected. All samples were collected with the informed consent of patients. The study was approved by the human ethics review committee of our hospital. Total RNA was extracted from samples using the TRIzol (Thermo Fisher Scientific). RNA was reverse transcribed into cDNA with Superscript II Reverse Transcriptase kit (Invitrogen) according to the supplier’s instructions. The gene expression was measured through qRT-PCR using SYBR Green PCR Master Mix kit (Invitrogen). The sequence of these primers was list in Table 1. Relative expression was calculated using 2^–ΔΔCt^ method, and GAPDH as a reference gene. The differences between two groups were compared by Student’s *t*-test. *P* < 0.05 (two-sided) was considered significant (Shan et al., 2020).

**TABLE 1:** Primers of hub genes.

**Genes**	**Primers**
GAPDH	F: 5′-CATGTTCGTCATGGGTGTGAA-3′
	R: 5′-GGCATGGACTGTGGTCATGAG-3′
FYN	F: 5′-ACCCATCCCGAACTACAC-3′
	R: 5′-CGCCAAACACAGTGTCACT-3′
KIF20A	F: 5′-TGTGGGTTTTCCCTGAGTTAGT-3′
	R: 5′-GATTTGGGGTCTGTGGTACG-3′
POLD1	F: 5′-GCTCCGCTCCTACACGCTCAA-3′
	R: 5′-GGTCTGGTCGTTCCCATTCTGC-3′
RAD54L	F: 5′-GAGCCCAGAGGACCTTGATA-3′
	R: 5′-AACCACCTTGTCTGGACAGC-3′
TYMS	F: 5′-GGGACTTGGGCCCAGTTTAT-3′
	R: 5′-CTTCTGTCGTCAGGGTTGGT-3′

### Single-Sample Gene Set Enrichment Analysis (ssGSEA)

The mark genes of immune cell types were collected from Bindea et al. (2013) and Zhang et al. (2020). The ssGSEA program was used to calculate the infiltration level of each immune cell. The correlation between hub genes and immune cell was calculated by Pearson correlation.

## Results

### DEGs in the Development of Infantile Hemangioma

**FIGURE 1:**
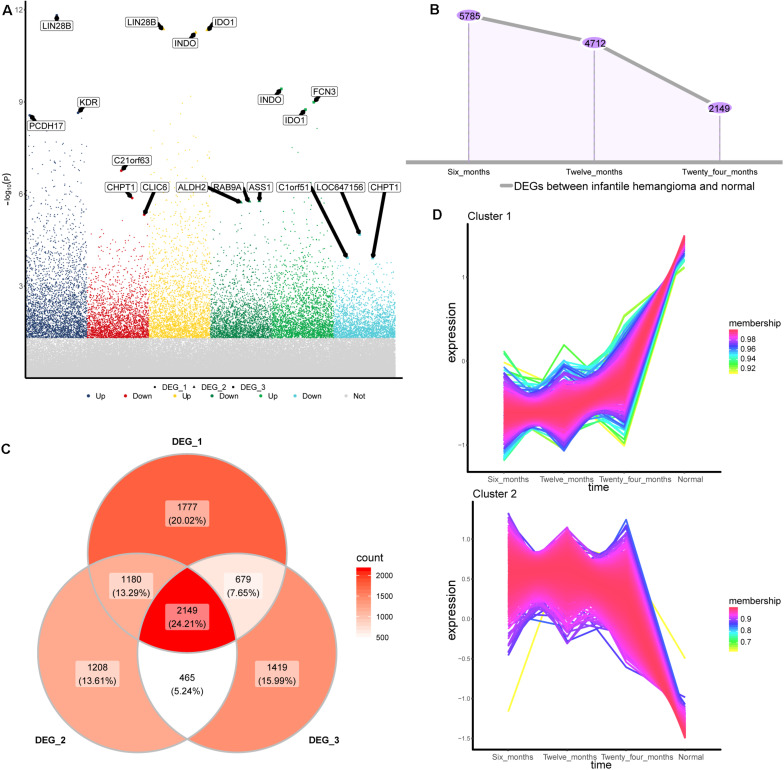
Differentially expressed genes between IH and normal. **(A)** The differentially expressed genes of hemangioma at 6, 12, and 24 months were compared with normal. Up: up-regulated; Down: down-regulated; Not: No changed significantly. **(B)** The numbers of differentially expressed genes in the three groups. **(C)** Venny map of differentially expressed genes for three groups. **(D)** Genes that are continuously up- or down-regulated of the common genes in STEM analysis. DEG_1: differentially expressed genes between 6 months and normal, DEG_2: differentially expressed genes between 12 months and normal, DEG_3: differentially expressed genes between 24 months and normal.

To identify the gene expression changes during the development of IH, we obtained differentially expressed genes (DEGs) between 6, 12, and 24 months hemangiomas and normal controls, respectively (Figure 1A). There were 5,785 DEGs between 6 months hemangioma and normal, 4,712 DEGs between 12 months hemangioma and normal, and 2,149 DEGs between 24 months hemangioma and normal. Interestingly, the numbers of DEGs gradually decrease over time (Figure 1B). Of these, 2,149 common DEGs were simultaneously present in the three groups of differences (Figure 1C). By STEM analysis of common genes, we obtained 1,218 genes that were continuously up- or down-regulated from 6 to 24 months and then to normal (Figure 1D).

### Co-expression Behavior of DEGs

**FIGURE 2:**
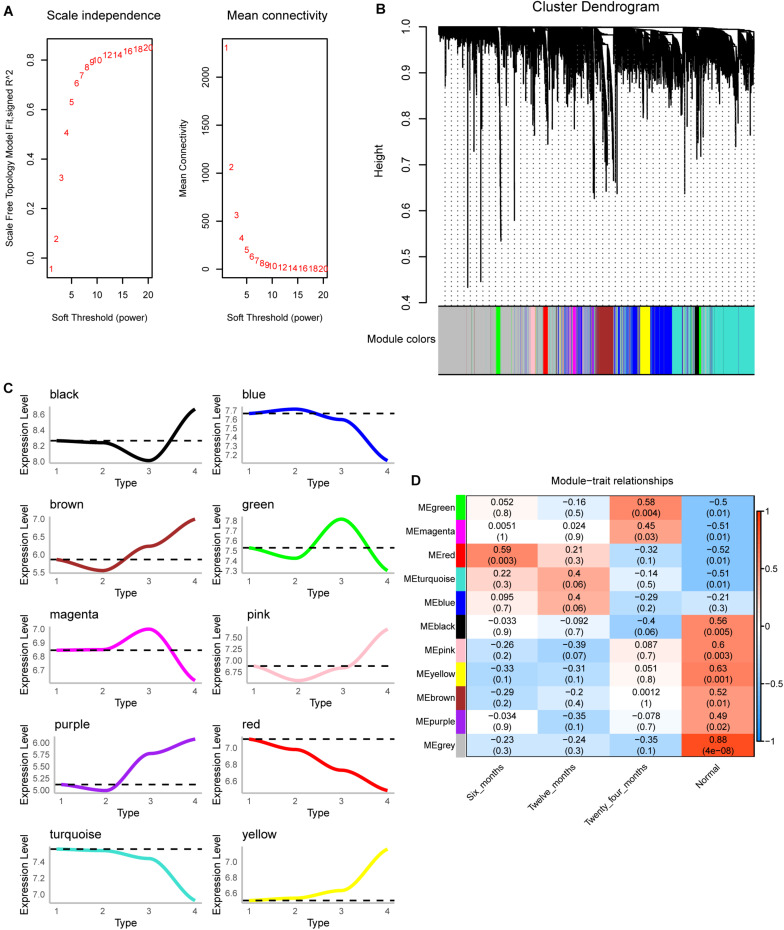
Co-expression analysis for differentially expressed genes of three groups. **(A)** The scale-free fit index and mean connectivity of WGCNA. **(B)** Differentially expressed genes were clustered into 10 modules. **(C)** The up-regulation or down-regulation of the module was calculated according to the average value of gene expression within the module. **(D)** Correlation between modules and clinical trait according to Pearson correlation.

The scale-free fit index and mean connectivity were calculated and the power of β = 9 (scale free *R*^2^ = 0.8) was selected (Figure 2A). WGCNA divided the union of DEGs of three groups into different modules. Finally, 10 modules were determined (Figure 2B). Moreover, we calculated the changing trend of genes in the module (Figure 2C). The expression of MEred and MEturquoise decreased with time, while that of MEyellow increased. Surprisingly, the correlation between module and trait showed that MEred (module 4) had the highest correlation with IH of 6 months, and then gradually decreased (Figure 2D). The correlation of MEyellow (module 3) increased gradually. We thought they were the most relevant modules to the development of IH.

### Biological Functions and Signaling Pathways of Module Genes

**FIGURE 3:**
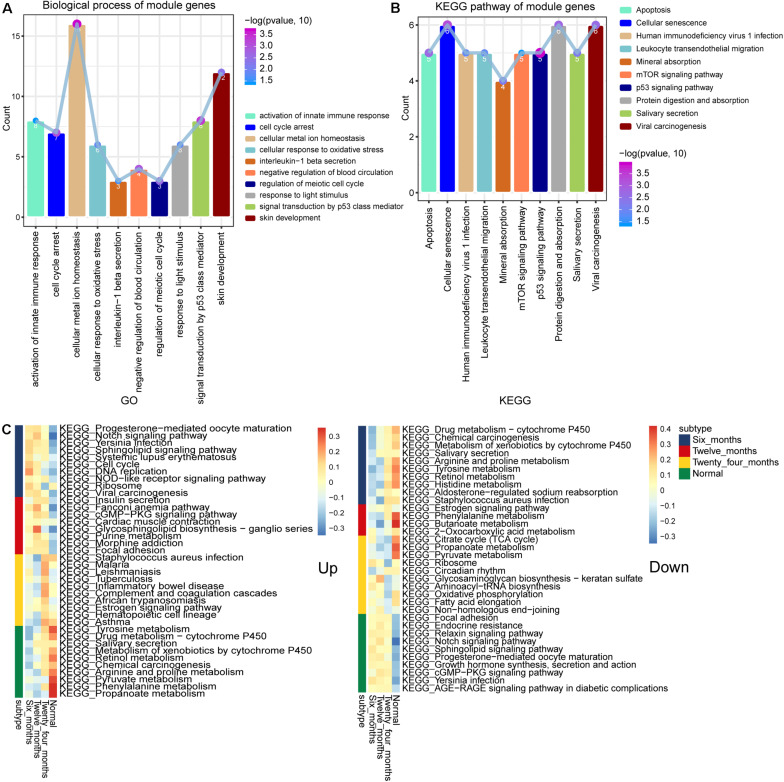
Biological functions and signaling pathways enriched by module genes. **(A)** The main biological processes of genes enriched in module 3 and module 4. **(B)** The main KEGG pathway of genes enriched in module 3 and module 4. **(C)** KEGG terms enriched in module genes that were continuously up- or down-regulated.

To further study the function of these module genes, GO, and KEGG analysis were performed. A total of 4,633 biological process (BP) terms, 562 cellular component (CC) terms and 995 molecular function (MF) terms were obtained. The DEGs in module 4 and module 3 were mainly enriched in activation of innate immune response, cell cycle arrest and cellular metal ion homeostasis (Figure 3A). In addition, the results of 264 KEGG terms were enriched for 10 module genes. The p53 signaling pathway, mTOR signaling pathway and Cellular senescence were included in module 4 and module 3 (Figure 3B). The results of subtypeGSEA found that Tyrosine metabolism, Retinol metabolism and Chemical carcinogenesis were gradually up-regulated with the improvement of IH, while Sphingolipid signaling pathway, Endocrine resistance and Notch signaling pathway were gradually down regulated (Figure 3C).

### Identification of Hub Genes

**FIGURE 4:**
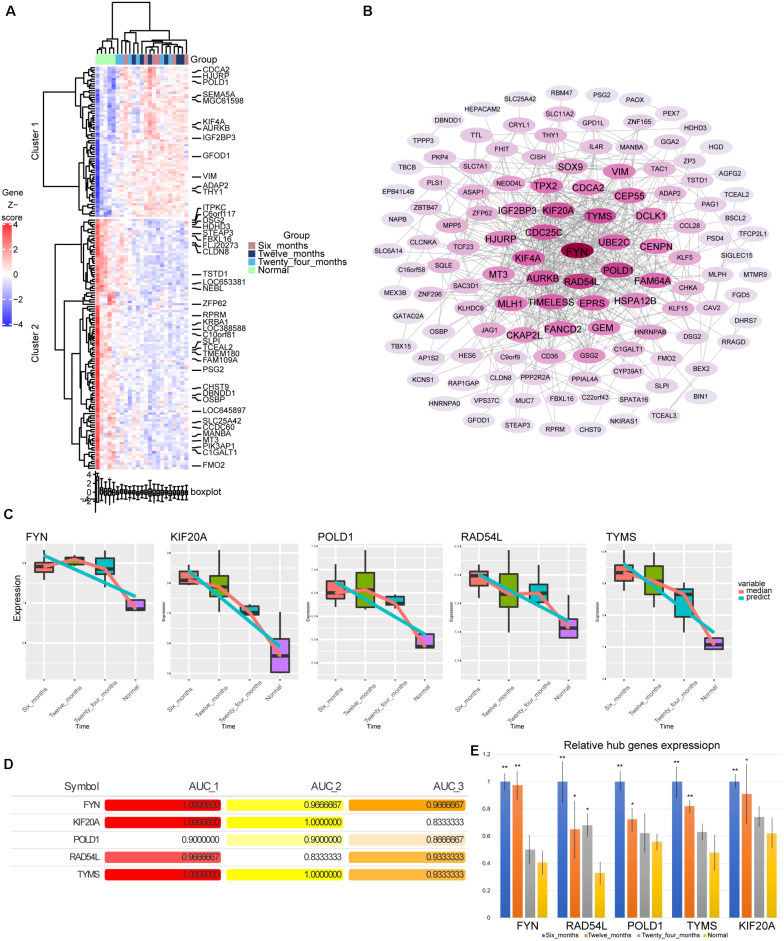
Screening of hub genes. **(A)** The heatmap of expression for176 genes. **(B)** The PPI network of 176 genes. The darker the color, the higher the connectivity of genes. **(C)** The expression level of hub genes in hemangioma at different time and normal group. **(D)** AUC values of hub genes at different stages. AUC_1: AUC values between 6 months and normal; AUC_2: AUC values between 12 months and normal; AUC_3: AUC values between 24 months and normal. **(E)** The relative expression level of hub genes in hemangioma at different stages detected by qRTPCR. *P < 0.05, **P < 0.01 were compared with normal.

By calculating the AUC values of the genes in modules 3 and 4 at different stages, we screened 176 genes with AUC values greater than 0.8 in all three groups. Most of these genes showed gradually up-regulated or down-regulated expression trend (Figure 4A). By constructing the PPI network, we identified the top five genes (FYN, KIF20A, POLD1, RAD54L, and TYMS) with the highest degree of connectivity in the network as hub genes (Figure 4B). Surprisingly, these five genes were also persistently dysregulated genes screened by STEM software. The hub genes were continuously down-regulated from 6 months to normal (Figure 4C). Their AUC values for hemangiomas at 6 months were higher than those at 24 months (Figure 4D). Fortunately, the differential expression of these genes was also validated by qRTPCR experiment (Figure 4E).

### Difference of Immune Infiltration in Hemangioma

**FIGURE 5:**
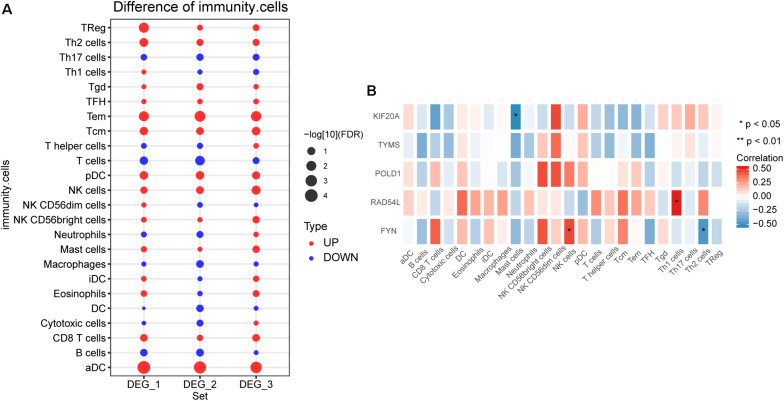
The immune cell infiltration between IHs and normal. **(A)** Differences in immune cell infiltration between hemangiomas and normal at different stages. DEG_1: differences in immune cell infiltration between 6 months and normal; DEG_2: differences in immune cell infiltration between 12 months and normal; DEG_3: differences in immune cell infiltration between 24 months and normal. **(B)** Correlation between immune cells and hub genes. *P < 0.05.

By comparing the difference of immune cell infiltration between hemangioma and normal at different stages, we found that Tem and aDC had the most significant difference among the three groups (Figure 5A). The results of correlation analysis showed that FYN was significantly positively correlated with NK cells, negatively correlated with Th2 cells (Figure 5B). KIF20A was negatively correlated with Mast cells, and RAD54L was positively correlated with Th1 cells.

## Discussion

IH is one of the most common tumors in children. Because of its complex etiology, the pathogenesis of hemangiomas remains unclear. This study was based on the different development stages of IH to explore the changes of gene expression during the development of the disease. The aim was to screen potential markers that could distinguish different stages of development. These genes were mainly associated with biological effects such as immune response, cell cycle, and apoptosis. The hub genes we obtained were significantly correlated with immune cells.

Interestingly, by comparing the number of DEGs between IH and normal at different stages, we found that the number of DEGs from the proliferative phase to the regressive phase was getting smaller and smaller. This also proved from the side that different time corresponded to different clinical periods. Nearly half of the genes in common DEGs were identified as persistently dysregulated by STEM. It suggested that these genes may be involved in the development of IH.

Co-expression analysis clusters genes with similar expression patterns into a co-expression module, each of which may characterize different molecular mechanisms (Lu et al., 2017). We found that module 4 had the highest correlation with the proliferative stage, followed by a gradual decline, and module 3 had the lowest correlation with the proliferative stage, followed by a gradual increase. These two module genes may be closely related to the development of IH. In fact, these two module genes were enriched in IH-related biological functions and signaling pathways.

The neonatal immune system is a developing structure that evolves in a complex, step-by-step manner (Basha et al., 2014). The innate immune defense system plays a key protective role in the growth of infants and young children (Yu et al., 2018). As expected, innate immunity is widespread in IH (Ritter et al., 2006; Peng et al., 2020). Promoting cell cycle arrest and apoptosis are the main mechanisms of action of drugs for the treatment of IH (Chen et al., 2019). In particular, p53 signaling pathway mediated apoptosis (Yao et al., 2018). The mammalian rapamycin complex (mTOR), which converts signals in the extracellular environment, such as glucose and growth factors, into corresponding changes in basic intracellular processes, including proliferation (Laplante and Sabatini, 2012). Rapamycin, an mTOR inhibitor, has anti-angiogenic effects on endothelial cells under pathological conditions (Guba et al., 2002).

During the development of IH, we found a large number of metabolic-related signaling pathways were continuously upregulated. Dysregulation of cell metabolism is listed as a new feature of cancer (Hirschey et al., 2015). Cellular metabolic pathways are targeted as central mediators of signaling and angiogenesis in health and disease (Wong et al., 2017). Our analysis results showed that the Notch signaling pathway was gradually down-regulated with the development of IH. Notch signaling pathway plays a key role in the development and progression of IH and is a potential target for the treatment of IH (Edwards et al., 2017).

The analysis results suggested that hub genes may had the ability to distinguish different stages of IH development. FYN is involved in many types of tumor progression, including hemangiomas (Llombart et al., 2009; van Oosterwijk et al., 2013; Panagopoulos et al., 2019, 2020). FYN regulates TLR4 signaling in mast cells and induces the secretion of tumor necrosis factor (Martin-Avila et al., 2016). KIF20A is a mitotic kinase that affects the prognosis of bladder cancer by promoting the proliferation and metastasis of bladder cancer cells (Sishtla et al., 2018; Shen et al., 2019). POLD1 is involved in controlling DNA repair and has been shown to be associated with cancer (Nicolas et al., 2016). High expression of POLD1 may serve as a potential prognostic indicator for invasive breast cancer (Qin et al., 2018). The cell cycle gene RAD54L may be inhibited by p53 to regulate G2/M cell cycle (Fischer et al., 2016). RAD54L was reported to be associated with cancer (Qi et al., 2013). TYMS was identified as a biomarker for hepatocellular carcinoma liver transplantation, pancreatic and colorectal cancer (Zhang et al., 2016; Ntavatzikos et al., 2017; Fu et al., 2019). However, the specific mechanism of action of these hub genes in IH is not yet clear.

This study also has some limitations. The analytical data we used were from public databases with a small sample size. To improve the reliability of data analysis results, we validated the differential expression of hub genes in IH and normal by qPCR experiments. In addition, the hub genes obtained in this study need further study to elucidate the discriminative ability of different IH stages and the relevant mechanism of action.

## Conclusion

In this study, bioinformatic methods were used to screen genes related to the development of infant hemangioma. We identified five key genes (FYN, KIF20A, POLD1, RAD54L, and TYMS) associated with the proliferative and regressive stages of IH. These key genes may regulate the development of IH through immune response, cell cycle, and mTOR pathway. Although our study was preliminary and further studies were needed to validate these findings. This network analysis based on WGCNA and PPI provided new insights into the diagnosis and treatment of IH patients.

## Data Availability Statement

The original contributions presented in the study are included in the article/supplementary material, further inquiries can be directed to the corresponding author/s.

## Author Contributions

XM: conception, design, and administrative support. All authors: provision of study materials or patients, collection, assembly of the data, the data analysis, interpretation, manuscript writing, and final approval of manuscript.

## Conflict of Interest

The authors declare that the research was conducted in the absence of any commercial or financial relationships that could be construed as a potential conflict of interest.

## Footnotes

1 https://string-db.org

## References

[B1] Basha S. Surendran N. Pichichero M. (2014). Immune responses in neonates. *Expert Rev. Clin. Immunol.* 10 1171–1184. 10.1586/1744666X.2014.942288 25088080 PMC4407563

[B2] Bindea G. Mlecnik B. Tosolini M. Kirilovsky A. Waldner M. Obenauf A. C. (2013). Spatiotemporal dynamics of intratumoral immune cells reveal the immune landscape in human cancer. *Immunity* 39 782–795. 10.1016/j.immuni.2013.10.003 24138885

[B3] Blei F. (2005). Basic science and clinical aspects of vascular anomalies. *Curr. Opin. Pediatr.* 17 501–509. 10.1097/01.mop.0000171322.76429.80 16012263

[B4] Boscolo E. Bischoff J. (2009). Vasculogenesis in infantile hemangioma. *Angiogenesis* 12 197–207. 10.1007/s10456-009-9148-2 19430954 PMC2810616

[B5] Botia J. A. Vandrovcova J. Forabosco P. Guelfi S. D’Sa K. United Kingdom Brain Expression Consortium (2017). An additional k-means clustering step improves the biological features of WGCNA gene co-expression networks. *BMC Syst. Biol.* 11:47. 10.1186/s12918-017-0420-6 28403906 PMC5389000

[B6] Chang L. C. Haggstrom A. N. Drolet B. A. Baselga E. Chamlin S. L. Garzon M. C. (2008). Growth characteristics of infantile hemangiomas: implications for management. *Pediatrics* 122 360–367. 10.1542/peds.2007-2767 18676554

[B7] Chaste P. Klei L. Sanders S. J. Hus V. Murtha M. T. Lowe J. K. (2015). A genome-wide association study of autism using the simons simplex collection: does reducing phenotypic heterogeneity in autism increase genetic homogeneity? *Biol. Psychiatry* 77 775–784. 10.1016/j.biopsych.2014.09.017 25534755 PMC4379124

[B8] Chen Z. Y. Wang Q. N. Zhu Y. H. Zhou L. Y. Xu T. He Z. Y. (2019). Progress in the treatment of infantile hemangioma. *Ann. Transl. Med.* 7:692. 10.21037/atm.2019.10.47 31930093 PMC6944559

[B9] Edwards A. K. Glithero K. Grzesik P. Kitajewski A. A. Munabi N. C. Hardy K. (2017). NOTCH3 regulates stem-to-mural cell differentiation in infantile hemangioma. *JCI Insight.* 2:e93764. 10.1172/jci.insight.93764 29093274 PMC5752265

[B10] Feltrin A. S. Tahira A. C. Simoes S. N. Brentani H. Martins D. C. Jr. (2019). Assessment of complementarity of WGCNA and NERI results for identification of modules associated to schizophrenia spectrum disorders. *PLoS One* 14:e0210431. 10.1371/journal.pone.0210431 30645614 PMC6333352

[B11] Fischer M. Quaas M. Steiner L. Engeland K. (2016). The p53-p21-DREAM-CDE/CHR pathway regulates G2/M cell cycle genes. *Nucleic Acids Res.* 44 164–174. 10.1093/nar/gkv927 26384566 PMC4705690

[B12] Fu Z. Jiao Y. Li Y. Ji B. Jia B. Liu B. (2019). TYMS presents a novel biomarker for diagnosis and prognosis in patients with pancreatic cancer. *Medicine* 98:e18487. 10.1097/MD.0000000000018487 31861032 PMC6940182

[B13] Gomez-Acevedo H. Dai Y. Strub G. Shawber C. Wu J. K. Richter G. T. (2020). Identification of putative biomarkers for infantile Hemangiomas and Propranolol treatment via data integration. *Sci. Rep.* 10:3261. 10.1038/s41598-020-60025-2 32094357 PMC7039967

[B14] Greenberger S. Adini I. Boscolo E. Mulliken J. B. Bischoff J. (2010a). Targeting NF-kappaB in infantile hemangioma-derived stem cells reduces VEGF-A expression. *Angiogenesis* 13 327–335. 10.1007/s10456-010-9189-6 20872175 PMC3034388

[B15] Greenberger S. Boscolo E. Adini I. Mulliken J. B. Bischoff J. (2010b). Corticosteroid suppression of VEGF-A in infantile hemangioma-derived stem cells. *N. Engl. J. Med.* 362 1005–1013. 10.1056/NEJMoa0903036 20237346 PMC2845924

[B16] Gu C. Shi X. Huang Z. Chen J. Yang J. Shi J. (2020). A comprehensive study of construction and analysis of competitive endogenous RNA networks in lung adenocarcinoma. *Biochim. Biophys. Acta Proteins Proteom.* 1868:140444 10.1016/j.bbapap.2020.140444 32423886

[B17] Guba M. von Breitenbuch P. Steinbauer M. Koehl G. Flegel S. Hornung M. (2002). Rapamycin inhibits primary and metastatic tumor growth by antiangiogenesis: involvement of vascular endothelial growth factor. *Nat. Med.* 8 128–135. 10.1038/nm0202-128 11821896

[B18] Hirschey M. D. DeBerardinis R. J. Diehl A. M. E. Drew J. E. Frezza C. Green M. F. (2015). Dysregulated metabolism contributes to oncogenesis. *Semin. Cancer Biol.* 35 (Suppl.), S129–S150. 10.1016/j.semcancer.2015.10.002 26454069 PMC4656121

[B19] Laplante M. Sabatini D. M. (2012). mTOR signaling in growth control and disease. *Cell* 149 274–293. 10.1016/j.cell.2012.03.017 22500797 PMC3331679

[B20] Liang W. Sun F. Zhao Y. Shan L. Lou H. (2020). Identification of susceptibility modules and genes for cardiovascular disease in diabetic patients using WGCNA analysis. *J. Diabetes Res.* 2020:4178639. 10.1155/2020/4178639 32455133 PMC7238331

[B21] Llombart B. Sanmartin O. Lopez-Guerrero J. A. Monteagudo C. Serra C. Requena C. (2009). Dermatofibrosarcoma protuberans: clinical, pathological, and genetic (COL1A1-PDGFB) study with therapeutic implications. *Histopathology* 54 860–872. 10.1111/j.1365-2559.2009.03310.x 19635106

[B22] Lu X. Lu J. Liao B. Li X. Qian X. Li K. (2017). Driver pattern identification over the gene co-expression of drug response in ovarian cancer by integrating high throughput genomics data. *Sci. Rep.* 7:16188. 10.1038/s41598-017-16286-5 29170526 PMC5700962

[B23] Luo Q. F. Zhao F. Y. (2011). The effects of Bleomycin A5 on infantile maxillofacial haemangioma. *Head. Face Med.* 7:11. 10.1186/1746-160X-7-11 21736714 PMC3148961

[B24] Mabeta P. (2018). Oncosuppressors and oncogenes: role in haemangioma genesis and potential for therapeutic targeting. *Int. J. Mol. Sci.* 19:1192. 10.3390/ijms19041192 29652858 PMC5979526

[B25] Martin-Avila A. Medina-Tamayo J. Ibarra-Sanchez A. Vazquez-Victorio G. Castillo-Arellano J. I. Hernandez-Mondragon A. C. (2016). Protein tyrosine Kinase Fyn Regulates TLR4-elicited responses on mast cells controlling the function of a PP2A-PKCalpha/beta signaling node leading to TNF secretion. *J. Immunol.* 196 5075–5088. 10.4049/jimmunol.1501823 27183589

[B26] Nicolas E. Golemis E. A. Arora S. (2016). POLD1: central mediator of DNA replication and repair, and implication in cancer and other pathologies. *Gene* 590 128–141. 10.1016/j.gene.2016.06.031 27320729 PMC4969162

[B27] Ntavatzikos A. Spathis A. Patapis P. Machairas N. Peros G. Konstantoudakis S. (2017). Integrating TYMS, KRAS and BRAF testing in patients with metastatic colorectal cancer. *World J. Gastroenterol.* 23 5913–5924. 10.3748/wjg.v23.i32.5913 28932083 PMC5583576

[B28] Panagopoulos I. Gorunova L. Lobmaier I. Andersen K. Lund-Iversen M. Micci F. (2020). Fusion of the COL4A5 gene with NR2F2-AS1 in a hemangioma carrying a t(X;15)(q22;q26) chromosomal translocation. *Cancer Genom. Proteom.* 17 383–390. 10.21873/cgp.20197 32576583 PMC7367601

[B29] Panagopoulos I. Gorunova L. Lobmaier I. Lund-Iversen M. Andersen K. Holth A. (2019). Fusion of the COL1A1 and FYN genes in epithelioid osteoblastoma. *Cancer Genom. Proteom.* 16 361–368. 10.21873/cgp.20141 31467230 PMC6727071

[B30] Peng H. Xian D. Liu J. Pan S. Tang R. Zhong J. (2020). Regulating the polarization of macrophages: a promising approach to vascular dermatosis. *J. Immunol. Res.* 2020:8148272. 10.1155/2020/8148272 32775470 PMC7407038

[B31] Qi L. N. Li L. Q. Chen Y. Y. Chen Z. H. Bai T. Xiang B. D. (2013). Genome-wide and differential proteomic analysis of hepatitis B virus and aflatoxin B1 related hepatocellular carcinoma in Guangxi, China. *PLoS One* 8:e83465. 10.1371/journal.pone.0083465 24391771 PMC3877066

[B32] Qin Q. Tan Q. Li J. Yang W. Lian B. Mo Q. (2018). Elevated expression of POLD1 is associated with poor prognosis in breast cancer. *Oncol. Lett.* 16 5591–5598. 10.3892/ol.2018.9392 30344713 PMC6176253

[B33] Reimand J. Isserlin R. Voisin V. Kucera M. Tannus-Lopes C. Rostamianfar A. (2019). Pathway enrichment analysis and visualization of omics data using g:Profiler, GSEA, Cytoscape and EnrichmentMap. *Nat. Protoc.* 14 482–517. 10.1038/s41596-018-0103-9 30664679 PMC6607905

[B34] Ritchie M. E. Phipson B. Wu D. Hu Y. Law C. W. Shi W. (2015). limma powers differential expression analyses for RNA-sequencing and microarray studies. *Nucleic Acids Res.* 43 e47. 10.1093/nar/gkv007 25605792 PMC4402510

[B35] Ritter M. R. Dorrell M. I. Edmonds J. Friedlander S. F. Friedlander M. (2002). Insulin-like growth factor 2 and potential regulators of hemangioma growth and involution identified by large-scale expression analysis. *Proc. Natl. Acad. Sci. U.S.A.* 99 7455–7460. 10.1073/pnas.102185799 12032304 PMC124252

[B36] Ritter M. R. Reinisch J. Friedlander S. F. Friedlander M. (2006). Myeloid cells in infantile hemangioma. *Am. J. Pathol.* 168 621–628. 10.2353/ajpath.2006.050618 16436675 PMC1606494

[B37] Shan G. Li X. Huang W. (2020). AI-enabled wearable and flexible electronics for assessing full personal exposures. *Innovation* 1:100031 10.1016/j.xinn.2020.100031 PMC8454737 34557709

[B38] Shen T. Yang L. Zhang Z. Yu J. Dai L. Gao M. (2019). KIF20A affects the prognosis of bladder cancer by promoting the proliferation and metastasis of bladder cancer cells. *Dis. Markers.* 2019:4863182. 10.1155/2019/4863182 31093305 PMC6481133

[B39] Shi X. Huang T. Wang J. Liang Y. Gu C. Xu Y. (2018). Next-generation sequencing identifies novel genes with rare variants in total anomalous pulmonary venous connection. *eBio Med.* 38 217–227. 10.1016/j.ebiom.2018.11.008 30448225 PMC6306349

[B40] Sishtla K. Pitt N. Shadmand M. O’Hare M. N. Sulaiman R. S. Sinn A. L. (2018). Observations on spontaneous tumor formation in mice overexpressing mitotic kinesin Kif14. *Sci. Rep.* 8:16152. 10.1038/s41598-018-34603-4 30385851 PMC6212535

[B41] Takahashi K. Mulliken J. B. Kozakewich H. P. Rogers R. A. Folkman J. Ezekowitz R. A. (1994). Cellular markers that distinguish the phases of hemangioma during infancy and childhood. *J. Clin. Invest.* 93 2357–2364. 10.1172/JCI117241 7911127 PMC294441

[B42] van Oosterwijk J. G. van Ruler M. A. Briaire-de Bruijn I. H. Herpers B. Gelderblom H. van de Water B. (2013). Src kinases in chondrosarcoma chemoresistance and migration: dasatinib sensitises to doxorubicin in TP53 mutant cells. *Br. J. Cancer* 109 1214–1222. 10.1038/bjc.2013.451 23922104 PMC3778302

[B43] Wong B. W. Marsch E. Treps L. Baes M. Carmeliet P. (2017). Endothelial cell metabolism in health and disease: impact of hypoxia. *EMBO J.* 36 2187–2203. 10.15252/embj.201696150 28637793 PMC5538796

[B44] Wu K. Q. Muratore C. S. So E. Y. Sun C. Dubielecka P. M. Reginato A. M. (2017). M1 macrophage-induced endothelial-to-mesenchymal transition promotes infantile hemangioma regression. *Am. J. Pathol.* 187 2102–2111. 10.1016/j.ajpath.2017.05.014 28710904 PMC5809337

[B45] Yao T. H. Pataer P. Regmi K. P. Gu X. W. Li Q. Y. Du J. T. (2018). Propranolol induces hemangioma endothelial cell apoptosis via a p53BAX mediated pathway. *Mol. Med. Rep.* 18 684–694. 10.3892/mmr.2018.9013 29767244 PMC6059697

[B46] Yu J. C. Khodadadi H. Malik A. Davidson B. Salles E. Bhatia J. (2018). Innate immunity of neonates and infants. *Front. Immunol.* 9:1759. 10.3389/fimmu.2018.01759 30105028 PMC6077196

[B47] Zhang H. M. Li S. P. Yu Y. Wang Z. He J. D. Xu Y. J. (2016). Bi-directional roles of IRF-1 on autophagy diminish its prognostic value as compared with Ki67 in liver transplantation for hepatocellular carcinoma. *Oncotarget* 7 37979–37992. 10.18632/oncotarget.9365 27191889 PMC5122365

[B48] Zhang L. Shi X. Gu C. Chen B. Wang M. Yu Y. (2020). Identification of cell-to-cell interactions by ligand-receptor pairs in human fetal heart. *Biochim. Biophys. Acta Mol. Basis Dis.* 1866:165917. 10.1016/j.bbadis.2020.165917 32800943

[B49] Zhu X. Guo X. Liu D. Gong Y. Sun J. Dong C. (2018). Significant inhibition of infantile hemangioma growth by sustained delivery of urea from liposomes-in-microspheres. *Oncol. Rep.* 39 109–118. 10.3892/or.2017.6103 29192323 PMC5783591

